# Identification and genetic characterization of *Sarcocystis arctica* and *Sarcocystis lutrae* in red foxes (*Vulpes vulpes*) from Baltic States and Spain

**DOI:** 10.1186/s13071-018-2694-y

**Published:** 2018-03-12

**Authors:** Viktorija Kirillova, Petras Prakas, Rafael Calero-Bernal, Inese Gavarāne, José Luis Fernández-García, Manuel Martínez-González, Eglė Rudaitytė-Lukošienė, Miguel Ángel Habela Martínez-Estéllez, Dalius Butkauskas, Muza Kirjušina

**Affiliations:** 1 Institute of Life Sciences and Technology, Daugavpils University, Parādes street 1A, Daugavpils, LV-5401 Latvia; 2 Laboratory of Molecular Ecology, Nature Research Centre, Akademijos 2, LT-08412 Vilnius, Lithuania; 3 Parasitology Section, Faculty of Veterinary Medicine, University of Extremadura, Avda. de la Universidad s/n, 10071 Cáceres, Spain; 4 SALUVET, Animal Health Department, Complutense University of Madrid, Avda. Complutense s/n, 28040 Madrid, Spain; 5 VPP AgroBioRes RISKI, Institute of Food Safety, Animal Health and Environment “BIOR”, Lejupes street 3, Riga, LV-1076 Latvia

**Keywords:** *Sarcocystis arctica*, *Sarcocystis lutrae*, Wild carnivores, Haplotypes, Phylogeny, Molecular characterisation

## Abstract

**Background:**

Typically, carnivores serve as definitive hosts for *Sarcocystis* spp. parasites; currently, their role as intermediate hosts is being elucidated. The present study aimed to identify and molecularly characterize *Sarcocystis* cysts detected in striated muscle of red foxes from different populations in Latvia, Lithuania and Spain.

**Methods:**

Muscle samples from 411 red foxes (*Vulpes vulpes*) and 269 racoon dogs (*Nyctereutes procyonoides*) from Latvia, 41 red foxes from Lithuania and 22 red foxes from Spain were examined for the presence of *Sarcocystis* sarcocysts by light microscopy (LM). *Sarcocystis* spp. were identified by transmission electron microscopy (TEM) and molecular biology techniques.

**Results:**

*Sarcocystis* cysts were detected in 11/411 (2.7%) Latvian, 3/41 (7.3%) Lithuanian, and 6/22 (27.3%) Spanish red foxes, however, cysts were not observed in the muscles of racoon dogs. Based on LM, TEM, *18S* rDNA, *28S* rDNA, ITS1, *cox*1 and *rpoB* sequences, *Sarcocystis arctica* and *Sarcocystis lutrae* cysts were identified in red fox muscles from Latvia and Lithuania, whereas only *S. arctica* was detected in Spain. The *18S* rDNA, *28S* rDNA and ITS1 sequences from the 21 isolates of *S. arctica* from Latvia, Lithuania and Spain were identical. By contrast, two and four haplotypes were determined based on mtDNA *cox*1 and apicoplast *rpoB* sequences, respectively. Polymorphisms were not detected between the two isolates of *S. lutrae* from Latvia and Lithuania. Based on phylogenetic results, *S. arctica* and *S. lutrae* were most closely related to *Sarcocystis* spp. using predatory mammals as intermediate hosts and to *Sarcocystis* species with a bird-bird life-cycle.

**Conclusions:**

Based on current knowledge, the red fox and Arctic fox (*Vulpes lagopus*) could act as intermediate host for the same two *Sarcocystis* species. Molecular results suggest the existence of two genetic lineages of *S. arctica*, and such divergence relies on its geographical distribution but not on their intermediate host species.

## Background

Protozoans of the genus *Sarcocystis* (Apicomplexa: Sarcocystidae) are worldwide distributed parasites of mammals, birds and reptiles. They are characterized by an obligatory two-host life-cycle, and their transmission is based on prey-predator relationships. Sarcocysts are mainly found in striated muscles of the intermediate host, and sporocysts develop in the small intestine of the definitive host. Some *Sarcocystis* species are clinically important for humans, domestic and wild animals; pathogenic effects such as abortions, weight loss, encephalitis, and myositis mainly occur in the intermediate hosts [1].

Initially, carnivores were considered the only definitive hosts of *Sarcocystis* spp.; however, over the last few years, numerous *Sarcocystis* species employing wild terrestrial carnivores as intermediate hosts have been described, namely *S. arctosi* from the brown bear (*Ursus arctos*) [2]; *S. ursuri* from the black bear (*Ursus americanus*) [3]; *S*. *kalvikus* and *S*. *kitikmeotensis* from the wolverine (*Gulo gulo*) [4]; *S. arctica* from the Arctic fox (*Vulpes lagopus*) [5] and *S. lutrae* from the Eurasian otter (*Lutra lutra*) [6]. The domestic dog usually acts as a definitive host within the genus *Sarcocystis*; however, dogs can also serve as intermediate hosts. Infections by *S. canis* and *S*. *neurona* have been reported in a wide variety of mammals, including dogs (reviewed by Dubey et al. [1]). Recently, two more species, *S. caninum* and *S. svanai* were described in domestic dogs [7]. The latter species have been genetically characterized by *18S* rDNA, ITS1 and *rpoB*. *Sarcocystis* species have relatively high host specificity with regards to their intermediate hosts [1]. Besides, reasonably high *Sarcocystis* infection rates have been reported in some carnivore species [8–11]. Thus, this data confirms that carnivores can also act as intermediate hosts of some *Sarcocystis* species.

The identification of *Sarcocystis* species is usually based on the morphology of sarcocysts, DNA sequences analysis and knowledge of parasite life-cycle [12]. Definitive hosts of recently described *Sarcocystis* species of wild carnivores remain unknown [1]. Comprehensive light microscopy (LM) and transmission electron microscopy (TEM) examination of sarcocysts of the majority *Sarcocystis* species from carnivores has been performed. Conversely, with the exception of *S*. *neurona*, *S. arctica* and *S. lutrae*, only limited data on DNA sequences of *Sarcocystis* species using mammal predators as intermediate hosts is available.

Recently, *S. arctica* was identified in muscles of red foxes (*Vulpes vulpes*) collected in the Czech Republic [13]. Here, we provide information on infection rates, morphological and molecular data of *Sarcocystis* sarcocysts detected in striated muscle of red foxes from different populations in Latvia, Lithuania and Spain.

## Methods

### Sample collection in Latvia

Between 2013 and 2016, hind leg muscle samples of 411 red foxes and 269 racoon dogs that were legally hunted, road-killed and trapped under state Rabies control and eradication programs were examined. Sampling covered the whole territory of Latvia and was uniformly distributed. Leg muscles were selected, for ease of access and availability of relatively large amounts of material.

### Sample collection in Lithuania

In 2015–2016, four muscle types, i.e. diaphragm, heart, hind leg and tongue of 42 red foxes legally hunted in the Central and East Lithuania were examined for *Sarcocystis* spp. infection under LM.

### Sample collection in Spain

Twenty-two red foxes, legally hunted in southwestern Spain in December 2016, were necropsied. Muscle tissue from tongue and forearms were morphologically examined by LM for the presence of sarcocysts.

### Light microscopy examination

*Sarcocystis* prevalence and infection intensity in animals collected from Latvia and Lithuania were evaluated in methylene blue-stained 28 oat-size fragments of muscle (~1 g) under LM using a previously described procedure [14]. In Spain, fresh-squashed muscle samples were examined for the presence of sarcocysts. After squeezing of fresh muscle tissues, sarcocysts were excised with the aid of preparation needles, and then morphologically characterized. The sarcocysts were differentiated according to the size and shape of the cyst, the structure of the cyst wall, and morphometric parameters of the bradyzoites. The isolated cysts were preserved in microcentrifuge tubes containing 96% ethanol and kept frozen at -20 °C for further examination by molecular methods. For molecular analyses, 11 cysts from 11 Latvian red foxes, 4 cysts from 3 Lithuanian red foxes (2 cysts from fox no. 24) and 8 cysts from 3 Spanish red foxes (4 cysts from fox no. 5; 2 cysts from fox no. 7 and 2 cysts from fox no. 11) were isolated.

In addition, sections of fresh muscle tissues from 1 Lithuanian and 22 Spanish foxes were buffered-formalin fixed prior to hematoxylin and eosin (H&E) staining of 5 μm thick and 2.5 cm^2^ sections.

### Electron microscopy examination

Four excised sarcocysts from Spanish foxes, and 1 from a Lithuanian fox were fixed in 2.5% glutaraldehyde and subjected to transmission electron microscopy (TEM) as reported previously [15, 16]. Briefly, samples were post-fixed in 1% buffered osmium tetroxide, and sections were cut on a Leica UC6 ultramicrotome and stained with 4% uranyl acetate and 3% lead citrate.

### Molecular analyses

Genomic DNA was extracted from individual sarcocysts using the QIAamp® DNA Micro Kit (Qiagen, Hilden, Germany) according to the manufacturer’s recommendations. *Sarcocystis* species were characterized at 5 loci, *18S* ribosomal DNA (rDNA), *28S* rDNA, ITS1 (internal transcribed spacer 1 region), *cox*1 (mitochondrial gene encoding subunit 1 of cytochrome *c* oxidase), and *rpoB* (RNA polymerase B gene of the apicoplast genome). The nearly complete *18S* rDNA sequences, partial *28S* rDNA sequences, complete ITS1 sequences, partial *cox*1 sequences and partial *rpoB* sequences were amplified using primers shown in Table 1.

**Table 1:** Primers used for the amplification of five DNA regions

DNA region	Primer name	Primer sequence (5′-3′)	Reference
*18S* rDNA	SarAF^a^	CTGGTTGATCCTGCCAGTAG	[30]
	SarBR^b^	GGCAAATGCTTTCGCAGTAG	[30]
	SarCF^a^	TTTAACTGTCAGAGGTGAAATTCTT	[30]
	SarDR^b^	GCAGGTTCACCTACGGAAA	[30]
*28S* rDNA	KL-P1F^a^	TACCCGCTGAACTTAAGCAT	[30]
	KL-P2R^b^	TGCTACTACCACCAAGATCTGC	[30]
ITS1	P-ITSF^a^	ATTACGTCCCTGCCCTTTGT	[30]
	P-ITSR^b^	GCCATTTGCGTTCAGAAATC	[30]
*cox*1	SF1^a^	ATGGCGTACAACAATCATAAAGAA	[31]
	SR5^b^	ATATCCATACCTCCATTGCCCAT	[31]
*rpoB*	RPObF^a^	TAGTACATTAGAAATCCCTAAAC	[32]
	RPObR^b^	TCWGTATAAGGTCCTGTAGTTC	[32]

^a^Forward primer

^b^Reverse primer

Each PCR reaction mixture contained 12.5 μl DreamTaq PCR Master Mix (2×) (Thermo Fisher Scientific Baltics, Vilnius, Lithuania), 0.05 μg template DNA, 1 μM of each primer, and nuclease-free water to constitute the final 25-μl volume. All amplification reactions were carried out with the same protocol: 5 min at 95 °C, 5 cycles of 45 s at 94 °C, 60 s at 64 °C, 90 s at 72 °C, followed by 30 cycles of 45 s at 94 °C, 60 s at 58 °C, and 70 s at 72 °C and 10 min at 72 °C. The PCR products were visualized using 1.7% agarose gel electrophoresis and purified with exonuclease ExoI and alkaline phosphatase FastAP (Thermo Fisher Scientific). The PCR products were sequenced directly with the 3500 Genetic Analyzer (Applied Biosystems, Foster City, CA, USA) using the same forward and reverse primers as for the PCR. The resulting sequences were edited manually when necessary and merged into single sequences representing each of the genetic regions investigated.

To search for highly similar sequences and determining sequence identity values, the newly obtained sequences were compared with those of various *Sarcocystis* spp. using the Nucleotide BLAST program (megablast option). Multiple alignments obtained using MUSCLE algorithm [17] were loaded into the MEGA7 software [18]. Phylogenetic relationships of the haplotypes were inferred by coalescent simulations using median-joining model implemented in NETWORK 5.0 [19, 20]. The TOPALi v2.5 software [21] was used to select a nucleotide substitution model with the best fit to the aligned sequences dataset and to construct the phylogenetic trees using the Bayesian inference algorithm.

## Results

### Infection rates and *Sarcocystis* species identification in red foxes from Latvia, Lithuania and Spain

Sarcocysts were detected in 11/411 Latvian red foxes (2.7%); cysts were not observed in the 294 racoon dogs examined in Latvia. The infection intensity of *Sarcocystis* spp. sarcocysts in red fox hunted in Latvia varied from 1 to 17 cysts (mean = 5.8, median = 4.0). Examination of diaphragm, heart, leg and tongue muscles of 41 red foxes hunted in Lithuania led to the identification of cysts found in 3 animals (7.3%). Cysts were observed in the hind leg and diaphragm muscle of 1 red fox (isolate NRS2) and only in the diaphragm muscles of two other red foxes (isolates NRS24 and NRS28). The intensity of infection was lower in Lithuanian samples (1 cyst/g in diaphragm muscle and 1–3 cysts/g in leg muscles). Finally, sarcocysts were detected in the forearm muscle tissues of 3 out of 22 red foxes (13.6%) hunted in Spain; however, prevalence of infection increased to 27.3% after examination of H&E-stained slides, with an average of 2.16 cysts/section (range 1–6; median = 1); cysts were not observed in tongue samples. Also, no foci of myositis were observed.

**Fig. 1:**
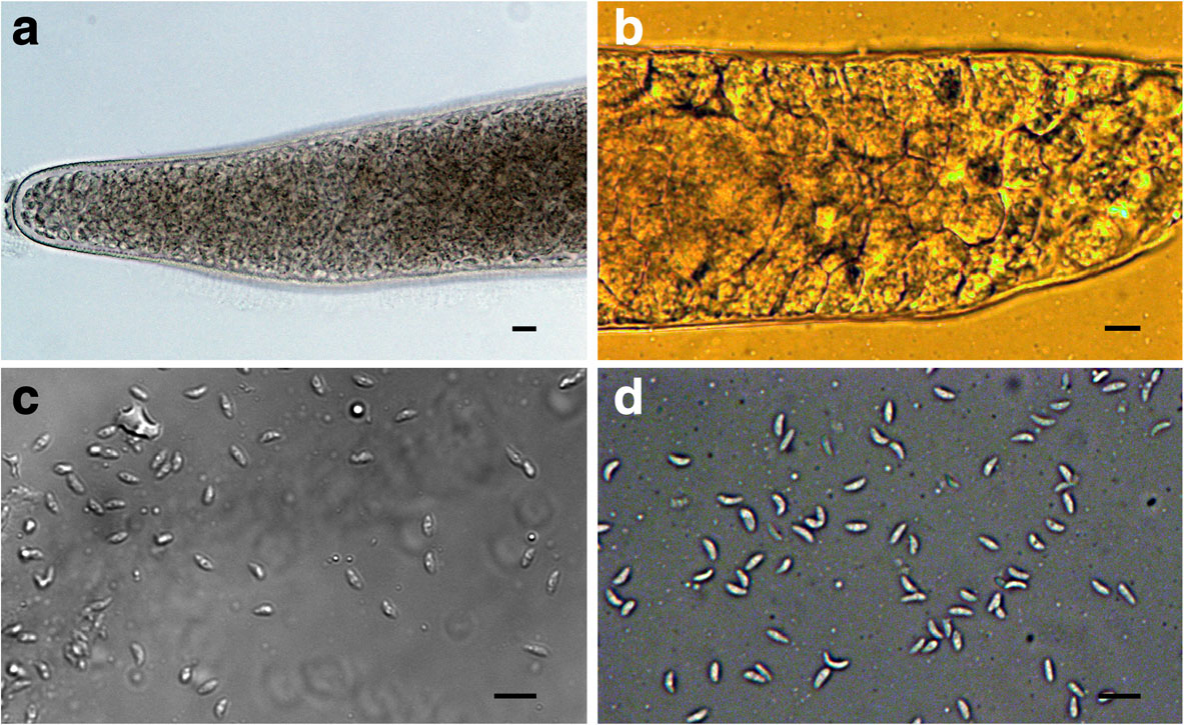
Light microscopy photomicrographs of *Sarcocystis arctica* (**a**, **c**) and *S. lutrae* (**b**, **d**) isolated from fresh muscle samples of the red fox (*Vulpes vulpes*). **a** Apical portion of sarcocyst; note knob-like villar protrusions on the cyst wall. **b** Portion of sarcocyst; note septae and thin, smooth wall with the apparent absence of villar protrusions. **c**, **d** Banana-shaped bradyzoites released from a broken sarcocysts. *Scale-bars*: 10 μm

**Fig. 2:**
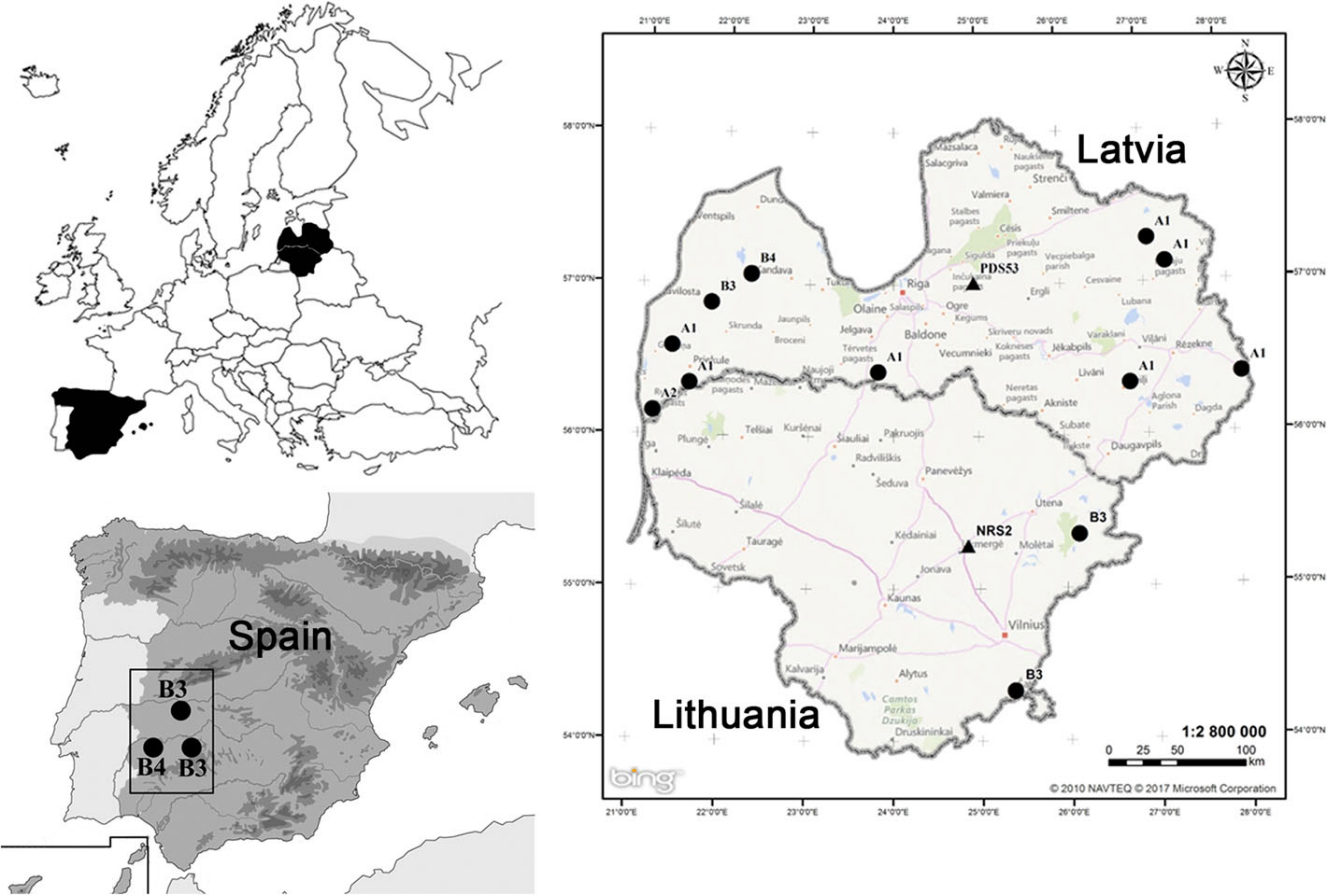
Distribution of *S. arctica* and *S. lutrae* in the sampled area and genotypes of *S. arctica* within *cox*1 and *rpoB*. Distribution of *S. arctica* is represented in *black circles* and *S. lutrae* in *black triangles*

Two morphological types of sarcocysts were observed in fresh muscle samples of red foxes with the aid of LM. Later, by molecular analysis, cysts were identified as *S. arctica* (Fig. 1a) and *S. lutrae* (Fig. 1b). Cysts of *S. arctica* were spindle-shaped, 2565–8414 × 83–204 μm in size, with rounded tip. The cyst wall was 1.6–2.8 μm thick and was characterised by knob-like protrusions on the surface. Septa divided the cyst into compartments, filled with banana-shaped bradyzoites, 6.5–9.3 × 1.6–2.4 (7.2 × 2.0) μm in size (Fig. 1c). Cysts of *S. lutrae* were spindle-shaped, and measured 1524–3186 × 75–111 μm, with a smooth cyst wall 0.8–2.2 μm thick. Banana-shaped bradyzoites measured 6.4–8.4 × 1.4–2.9 (7.1 × 2.0) μm (Fig. 1d). It should be noted that only one species, either *S. arctica* or *S. lutrae*, was detected in each infected animal. Based on the comparison of DNA sequences obtained, 10 foxes from Latvia, 3 from Spain and 2 from Lithuania harboured *S. arctica*, whereas 1 fox from Latvia and 1 from Lithuania were infected by *S. lutrae*. Two isolates of *S. lutrae* were detected in foxes hunted in Central Latvia and Central Lithuania (Fig. 2).

### Light and electron microscopy examination

**Fig. 3:**
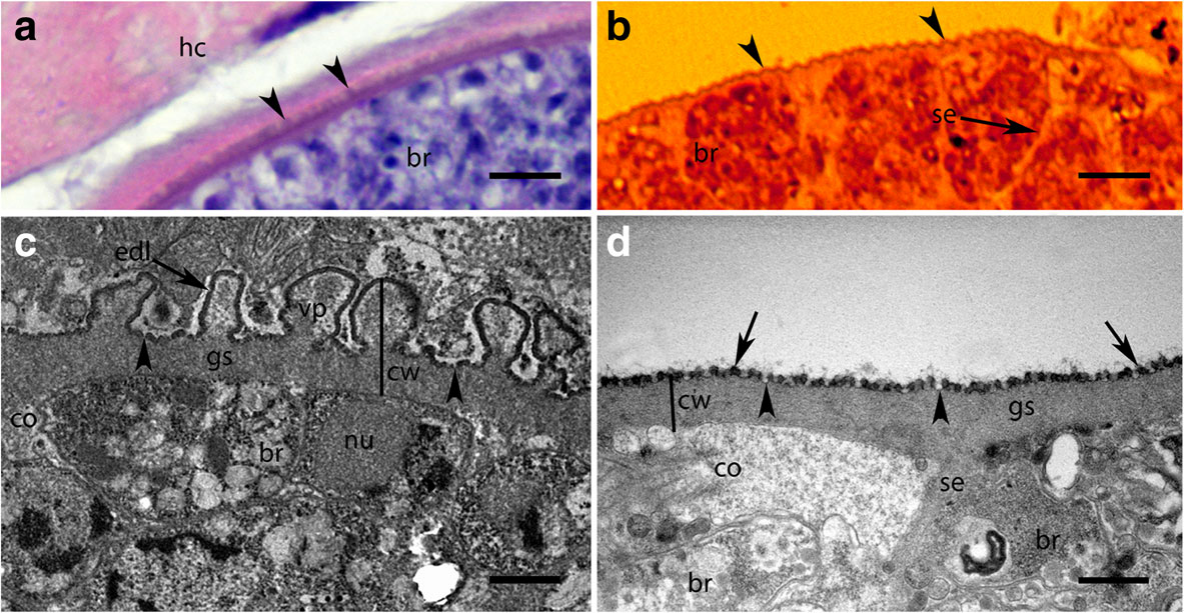
Comparison of light (LM) and transmission electron microscopy (TEM) photomicrographs of *Sarcocystis arctica* (**a**, **c**) and *S. lutrae* (**b**, **d**) from the red fox (*Vulpes vulpes*). **a** LM micrograph showing thin-walled cyst, with barely defined villar protrusions (vp, arrowheads); note bradyzoites (br) and muscular host cell (hc). H&E-staining. **b** LM micrograph showing well-defined thin cyst wall, with minute undulation due to fixation (arrowheads); note bradyzoites (br) and septum (se). H&E-staining. **c** TEM micrograph showing details of villar protrusions (vp), the total width of cyst wall (cw), ground substance (gs) with few electron-dense granules, and invaginations (arrowheads) of the electron dense layer (edl); note, details of bradyzoites (br), nucleus (nu) and conoid (co). **d** TEM micrograph is showing details of the simplest structure among cyst walls types within genus *Sarcocystis*, smooth ground substance (gs), blebs (arrows), invaginations (arrowheads), septum (se) and bradyzoite (br) with defined microtubules in conoid (co). *Scale-bars*: **a**, **b**, 10 μm; **c**, **d**, 1 μm

On H&E-stained sections, *S. arctica* sarcocysts are slender, thin-walled, and present minute and indistinct protrusions (arrowheads) (Fig. 3a); *S. lutrae* sarcocysts are slender, thin-walled (< 1 μm), and presented well-defined septae (se) grouping bradyzoites (Fig. 3b). By TEM, the *S. arctica* cyst wall closely resembles type 9c (Fig. 3c); the villar protrusions (vp) were up to 1.3 μm long and 0.5 μm wide; some vp appear anastomosing; the electron-dense layer (edl) had invaginations distributed at irregular distances (arrowheads). The cyst wall (cw) was ~ 1.7–1.8 μm-thick, and the cyst contained up to 6.4 μm-long (*n* = 5) mature bradyzoites (br). In about 0.7 μm-thick ground substance (gs) very few electron-dense granules were observed. Also, by TEM, *S. lutrae* cyst wall displayed a simple structure resembling type 1a (Fig. 3d). The parasitophorous vacuolar membrane is about 120 nm-thick and is characterised by a wavy outline, small knob-like blebs with rounded ends (arrows), and the edl also presents invaginations (arrowheads). The cyst wall is up to 0.8 μm in thickness. Ground substance is smooth and projects septae (se) that surround mature up to 6.0 μm-long (*n* = 3) bradyzoites.

### Molecular characterisation of *S. arctica*

**Fig. 4:**
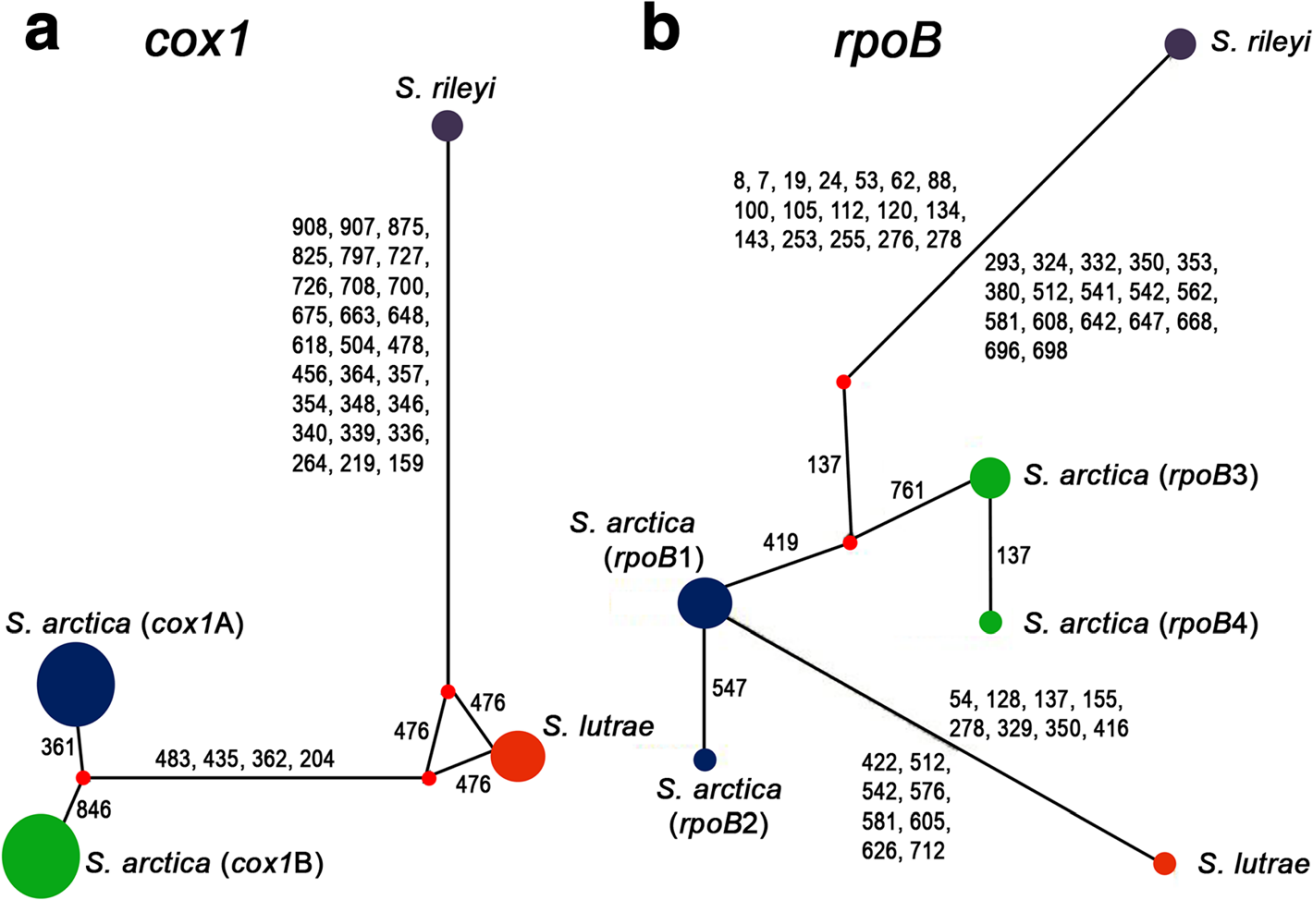
Haplotype networks obtained from *cox*1 (**a**) and *rpoB* (**b**), respectively. The numbers show the mutation sites splitting haplotypes. Small red dots indicate median vectors which represent hypothesized (often unsampled or extinct ancestral) sequence [20]. Haplotypes of *S. arctica* are shown in brackets. Blue and green dots indicate different *S. arctica* haplotypes; same colour groups 1 singleton difference

The *18S* rDNA (1803 bp long), *28S* rDNA (1468 bp), ITS1 (697 bp), *cox*1 (1053 bp) and *rpoB* (762 bp) sequences of *S. arctica* were deposited in GenBank under the accession numbers MF596217–MF596237, MF596240–MF596260, MF596262–MF596282, MF596286–MF596306 and MF596311–MF596331, respectively. Cysts that were isolated from the same animal showed no genetic differences at five loci analysed. The 21 isolates of *S. arctica* from Latvia, Lithuania and Spain showed 100% sequence identity among each other for *18S* rDNA, *28S* rDNA and ITS1 sequences. By contrast, 2 distinct haplotypes A and B, differing by 2 SNPs, were determined based on *cox*1 sequence analysis. In particular, nucleotide A was found in 439 and 924 segregating sites for haplotype A, while nucleotides T and C were identified at the same sites for haplotype B, respectively. Four polymorphic sites were observed within *rpoB* (Fig. 4), 2 of which were singleton variable sites (137 and 547) and the other 2 were parsimony informative sites (419 and 761). Four different haplotypes (haplotype 1, 2, 3 and 4) of *S. arctica* were determined based on *rpoB* sequence analysis. The haplotypes differed from each other by 1–4 substitutions. One mutation was observed between haplotype 1 and haplotype 2, and between haplotype 3 and haplotype 4. Haplotypes 1 and 2 differed from haplotypes 3 and 4 by 3 SNPs on average. Interestingly, based on concatenated *cox1* and *rpoB* sequences, only 4 different genotypes were established (A1, A2, B3 and B4), i. e. isolates having haplotype A within *cox*1 carried haplotypes 1 or 2 within *rpoB,* while isolates having haplotype B within *cox*1 alternatively presented haplotypes 3 and 4 within *rpoB* (Fig. 4).

Four genotypes of *S. arctica* were identified in Latvia and Lithuania, and their distribution in these 2 neighbouring countries was sporadic, while in Spain only genotypes B3 and B4 were detected (Fig. 2).

Isolates of *S. arctica* from red foxes in Latvia, Lithuania and Spain were compared with those from the Czech Republic, Norwegian and Alaskan Arctic foxes, and Alaskan wolf (*Canis lupus*) from the USA at the *18S* rDNA, *28S* rDNA, ITS1 and *cox*1 genetic loci. Based on *28S* rDNA and ITS1 sequences, *S. arctica* showed 99.9–100% and 99.2–100% identity, respectively. Two *S. arctica* haplotypes were identified in *cox1*, whereas this species had no intraspecific variability at *18S* rDNA.

Based on *18S* rDNA sequences, *S. arctica* was identical to *S*. *caninum* (GenBank: KM362427) from the domestic dog (*Canis familiaris*), 99.5% similar to *S*. *svanai* (GenBank: KY292487) from the domestic dog, 99.4% similar to *S*. *columbae* (GenBank: GU253883) from the wood pigeon (*Columba palumbus*), and displayed a very high sequence identity to other *Sarcocystis* spp. using predatory mammals and birds as intermediate hosts. At *28S* rDNA, *S. arctica* was most similar to *Sarcocystis* sp. ex *Accipiter cooperii* (GenBank: KY348754), *S*. *lari* (GenBank: JQ733509) and *S*. *calchasi* (GenBank: FJ232949), infecting birds of the orders Columbiformes and Psittaciformes (approximately 98–99% sequence identity). The newly obtained ITS1 sequences of *S. arctica* showed 99.5% identity to sequences of *S. caninum* isolated from muscles of the Rottweiler dog (GenBank: JX993923, JX993924; case C in [7]) and only 88% similarity with *S. felis* (GenBank: AY190081); even lower rates were observed with sequences from other *Sarcocystis* spp. employing predatory mammals as intermediate hosts including *S. canis*, *S*. *kalvikus* and *S. lutrae*. The *cox*1 sequences of *S. arctica* showed > 99% similarity to sequences from *S. lutrae*, *S*. *lari*, *S. canis* and several other *Sarcocystis* spp. Interestingly, the 462 bp long *rpoB* sequence of *S*. *caninum* (GenBank: KC191641) was identical with haplotype 1 of *S. arctica* and differed by one SNP (C/T) from other three *S. arctica* haplotypes determined in this study. Sequences of *rpoB* of *S. arctica* also showed identity to *S*. *lari* (GenBank: MF596307; 98.2–98.4%), *S. campestris* (GenBank: GQ851963) from ground squirrels (Sciuridae) (98.1–98.4%), *S. lutrae* (GenBank: MF596309; 97.8–97.9%), *S*. *svanai* from the domestic dog (GenBank: KC191641; 97.6–97.8%) and *S. canis* (GenBank: KC191642) from the polar bear (*Ursus maritimus*) (97.4–97.6%).

### Molecular characteristics of *S. lutrae*

Both isolates of *S. lutrae* from Latvia (PDS53) and Lithuania (NRS2) had identical *18S* rDNA (1782 bp) (MF596215, MF596216), *28S* rDNA (1492 bp) (MF596238, MF596239), *cox*1 (1053 bp) (MF596284, MF596285) and *rpoB* (762 bp) (MF596309, MF596310) sequences. The newly obtained *S. lutrae* sequences of *18S* and *28S* rDNA showed 100% identity with those of *S. lutrae* from the Eurasian otter detected in Norway. Based on *cox*1, *S. lutrae* from the red fox had 100% identity with *S. lutrae* from the Eurasian otter (GenBank: KM657808, KM657809) and the Arctic fox (GenBank: KF601326, KF601327). Unfortunately, only 929 bp of ITS1 of *S. lutrae* (GenBank: MF596261) from Lithuania were obtained, while ITS1 amplification using the *S. lutrae* isolate from Latvia was unsuccessful. The ITS1 sequence of *S. lutrae* from the red fox showed 100% identity with *S. lutrae* from the European badger (*Meles meles*) (GenBank: KX431307) from Scotland and had 98.0–100% identity with *S. lutrae* from the Eurasian otter (GenBank: KM657773–KM657805) studied in Norway.

Based on *18S* rDNA, *S. lutrae* had very high (exceeding 99%) sequence identity with several *Sarcocystis* species. The *28S* rDNA sequences of *S. lutrae* was 99.4% similar to *Sarcocystis* sp. ex *Accipiter cooperii* (GenBank: KY348754), 98.3% similar with *S*. *lari* (GenBank: JQ733509), and 98.2% similar with *S*. *turdusi* (GenBank: JF975682) from thrushes. Based on ITS1, *S. lutrae* was characterized by the lowest sequence differences with *S*. *kalvikus* (GenBank: GU200661; 3.8–5.2%). Interestingly, at *cox*1 *S. lutrae* demonstrated 100% sequence identity with *S*. *lari* (GenBank: MF596283) and differed only by one SNP from *Sarcocystis* sp. ex *Accipiter cooperii* (GenBank: KY348756) and *S*. *calchasi* (GenBank: KU220952). At *rpoB*, *S. lutrae* had the highest sequence similarity to *S*. *svanai* (GenBank: KC191641; 98.5%), *S. campestris* (GenBank: GQ851963; 97.7%), *S*. *caninum* (GenBank: KC191641; 97.6%), *S. arctica* (GenBank: MF596311– MF596331; 97.8–97.9%) and *S*. *lari* (GenBank: MF596307; 97.5%).

### Phylogeny

**Fig. 5:**
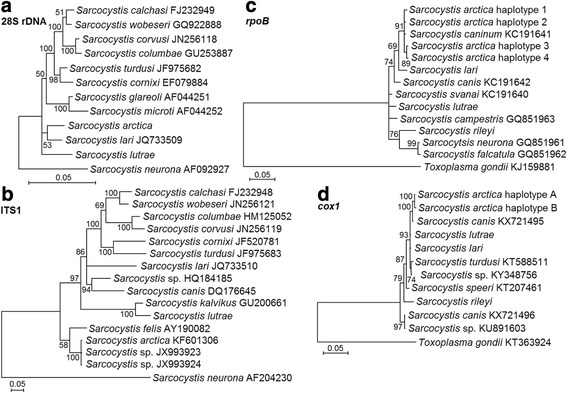
The phylogenetic placement of *S. arctica* and *S. lutrae* based on *28S* rDNA (**a**), ITS1 (**b**), *rpoB* (**c**) and *cox*1 (**d**) sequences*.* Sequences for *Sarcocystis* sp. (GenBank: JX993923, X993924) correspond to *S*. *caninum* [7]

Phylogenetic results showed that both species identified in the red fox, *S. arctica* and *S. lutrae*, were most closely related to *Sarcocystis* spp. using predatory mammals as intermediate hosts and *Sarcocystis* species characterized by a bird-bird life-cycle (Fig. 5). Phylogenetic analyses demonstrated that *S. arctica* could not be discriminated from *S*. *caninum* using *rpoB* and ITS1 sequences. However, based on *28S* rDNA and *cox*1 sequences, *S. arctica* was grouped with *S*. *lari* and *S. canis*, respectively. It should be noted that *28S* rDNA and *cox*1 sequences of *S*. *caninum* are not available on GenBank. The exact phylogenetic position of *S. lutrae* using *28S* rDNA, *cox*1 and *rpoB* sequences remains unclear. Based on ITS1, the most variable locus analysed here, *S. lutrae* was the sister species to *S*. *kalvikus*, which both infect mustelids.

## Discussion

The racoon dog is naturally distributed in the east of Asia. In the mid-twentieth century, one of the five racoon subspecies, *Nyctereutes procyonoides ussuriensis* was introduced in eastern Europe and nowadays is successfully expanding to the central part of the continent [22].

In the present study, hind leg muscles of 294 racoon dogs were negative for sarcocysts in Latvia. However, muscular *Sarcocystis* sp. infection was previously reported in the Japanese racoon dog (*N. p. viverrinus*). In Japan, sarcocysts were detected in 12 out of 27 animals examined (44.4%) [9]. Prevalence of the *Sarcocystis* infection varied significantly in different muscles examined in this study and was highest in the tongue (47.6%) and diaphragm (44.4%), the masseter muscles (28.0%), and lowest in the heart (14.8%) and oesophagus (4.0%). Furthermore, the severe meningoencephalitis associated with *Sarcocystis* sp. asexual development was reported in a free-living, adult racoon dog from Japan [23]. Further studies examining different types of muscle and covering broader geographical regions are needed to verify whether racoon dog populations, located far from their native range, could harbour muscular *Sarcocystis* infections.

The red fox is one of the main predators of the Canidae in the Northern Hemisphere [24]. In the present study, *S. arctica* and *S. lutrae* in red foxes from Latvia, Lithuania and Spain were characterized using LM and TEM, and DNA sequences analysis within five loci. Cysts of *Sarcocystis* spp. were first found in the muscles of the red fox in Kazakhstan [25]. Briefly, by LM, two morphological types of sarcocyst were distinguished. Cysts (1300–3900 × 130–500 μm in size) having a 2.0–2.8 μm thick and striated cyst wall were named *Sarcocystis vulpis*. Smaller cysts (25–520 × 40–65 μm in size), with smooth 1.4–2.1 μm thick cyst wall were assigned to *Sarcocystis* sp. However, in the latest taxonomic review of the genus *Sarcocystis*, *S*. *vulpis* was considered to be *species inquirenda* [1]. Based on morphological characters, the proposed *S*. *vulpis* and *Sarcocystis* sp. in red foxes from Kazakhstan [25] should correspond to *S. arctica* and *S. lutrae*, respectively. In 2009, *Sarcocystis* sp. cysts were found in one Japanese red fox (*Vulpes vulpes japonica*) [9]. By LM, cysts were narrow and short (21–710 × 20–47 μm) and by TEM cyst wall was thin (0.4–0.5 μm thick) and had minute undulations. Morphologically, *Sarcocystis* sp. from the Japanese red fox [9] is similar to *S. lutrae*. Recently, the Arctic fox and the Alaskan wolf were identified as being an intermediate host of *S. arctica* [5, 26, 27]. Moreover, based on *cox*1 comparison it was shown that one Arctic fox from Norway acted as the intermediate host for two *Sarcocystis* species, *S. arctica* and *S. lutrae* [6].

Here, using LM, *S. arctica* and *S. lutrae* show thin-walled cysts that can be distinguished within the same host (Fig. 3a, b). *Sarcocystis arctica* detected in the red fox here presents similar ultrastructure as previously described in the Alaskan wolf and Arctic fox [26, 27] in North America. In addition, *S. lutrae* sarcocysts from red foxes are morphologically similar to those of other *Sarcocystis* species detected in carnivores, e.g. *S. kalvikus* from the wolverine [4], *S. arctosi* from the brown bear [2], and *S. svanai* from a domestic dog [7] and the Pampax fox (*Lycalopex gymnocercus*) from Argentina [11]. Recently, *S. lutrae* has been detected in the European badger from Scotland [28], but no ultrastructural details were provided. Definitive hosts of *S. arctica* and *S. lutrae* remain unknown.

Based on data collected thus far, *S. lutrae* shows intraspecific diversity only within ITS1, while *S*. *arctica* is characterized by some intraspecific variability within *28S* rDNA, ITS1, *cox*1 and *rpoB*. The clearly distinguished *S. arctica* haplotypes A and B at *cox*1 were identified for Norwegian isolates of the Arctic fox, and for Latvian/Lithuanian and Czech isolates from the red fox, whereas Alaskan isolates from two intermediate hosts, the grey wolf and Arctic fox, were only haplotype A, and *S. arctica* from the Spanish red fox had haplotype B ([5, 13, 26, 27] and present study). At *rpoB*, two SNPs were determined between haplotype 1 and 3, and only one SNP between haplotype 1 and 2, and between haplotype 3 and 4 (Fig. 4). Primary population genetic data suggest the existence of two genetic lines of *S. arctica* expanding along the latitudinal cline. Distinguished genetic lines might be associated to different intermediate hosts. However, it is not possible to explain such diversity by historical geographical barriers given the fact that the host *Vulpes vulpes* is highly vagile and evenly distributed. Thus, the hypothesis that this particular genetic diversity within *S. arctica* species obeys to a potential adaptive significance [29] cannot be rejected.

## Conclusions

In the present study, sarcocysts of *S. arctica* and *S. lutrae* infecting red foxes from three different European countries were morphologically and molecularly characterized. Muscle samples of racoon dogs were also investigated, but no sarcocysts were found. LM and TEM provided details to distinguish both *Sarcocystis* species. Based on nuclear *18S* rDNA, *28S* rDNA, and ITS1 sequences, the 21 isolates of *S. arctica* from Latvia, Lithuania and Spain showed a 100% sequence identity among each other. However, 2 and 4 haplotypes were determined within *cox*1 and *rpoB*, respectively. Polymorphisms were not detected between the 2 isolates of *S. lutrae* studied herein. In conclusion, it is being demonstrated that red fox acts as an intermediate host for at least 2 species of *Sarcocystis*, and it is suggested that the existence of 2 genetic lineages of *S. arctica*, along with molecular divergence of such species relies on its geographic distribution.

## Abbreviations

br: bradyzoites
co: conoid
*cox*1: cytochrome *c* oxidase subunit 1 gene
cw: cyst wall
edl: electron-dense layer
gs: ground substance
H&E: hematoxylin and eosin staining
hc: muscular host cell
ITS1: internal transcribed spacer 1
LM: light microscopy
mtDNA: mitochondrial deoxyribonucleic acid
nu: nucleus
*rpoB*: RNA polymerase beta-subunit gene
se: septum
SNPs: single nucleotide polymorphisms
TEM: transmission electron microscopy
vp: villar protrusions
